# Modular Design of Picroside-II Biosynthesis Deciphered through NGS Transcriptomes and Metabolic Intermediates Analysis in Naturally Variant Chemotypes of a Medicinal Herb, *Picrorhiza kurroa*

**DOI:** 10.3389/fpls.2017.00564

**Published:** 2017-04-11

**Authors:** Varun Kumar, Ankush Bansal, Rajinder S. Chauhan

**Affiliations:** Department of Biotechnology and Bioinformatics, Jaypee University of Information TechnologyWaknaghat, India

**Keywords:** correlation, metabolic flux, picrosides, *Picrorhiza kurroa*, NGS transcriptomes

## Abstract

Picroside-II (P-II), an iridoid glycoside, is used as an active ingredient of various commercial herbal formulations available for the treatment of liver ailments. Despite this, the knowledge of P-II biosynthesis remains scarce owing to its negligence in *Picrorhiza kurroa* shoots which sets constant barrier for function validation experiments. In this study, we utilized natural variation for P-II content in stolon tissues of different *P. kurroa* accessions and deciphered its metabolic route by integrating metabolomics of intermediates with differential NGS transcriptomes. Upon navigating through high vs. low P-II content accessions (1.3–2.6%), we have established that P-II is biosynthesized *via* degradation of ferulic acid (FA) to produce vanillic acid (VA) which acts as its immediate biosynthetic precursor. Moreover, the FA treatment *in vitro* at 150 μM concentration provided further confirmation with 2-fold rise in VA content. Interestingly, the cross-talk between different compartments of *P. kurroa*, i.e., shoots and stolons, resolved spatial complexity of P-II biosynthesis and consequently speculated the burgeoning necessity to bridge gap between VA and P-II production in *P. kurroa* shoots. This work thus, offers a forward looking strategy to produce both P-I and P-II in shoot cultures, a step toward providing a sustainable production platform for these medicinal compounds via-à-vis relieving pressure from natural habitat of *P. kurroa*.

## Introduction

*Picrorhiza kurroa*, locally named as Kutki, is a high altitude (3000–5000 m) medicinal herb distributed in the North-Western Himalayas of India. It is used for the treatment of various ailments due to pharmacological properties like hepatoprotective, antiallergic, antiasthmatic, antioxidant, anticancerous, and immunomodulatory and therefore, consequently provide livelihood and health security to a large segment of high altitude populations (Kumar et al., 2015a, 2017). It is listed among top 15 most traded medicinal herbs in India with respect to revenues generated by traded material (Uniyal et al., 2011). *P. kurroa* is a self-propagating medicinal herb. It originates as a young bud on stolons which develops to produce mature stolon and subsequently rhizomes with self-governing roots and shoots compartments (Pandit et al., 2013a). Thus, during the collection of mature rhizomes of *P. kurroa*, all the young stolons and buds are also get uprooted which subsequently disrupt it’s proliferation in natural habitat. The extensive harvesting has thus put this herb in the category of critically endangered medicinal herbs and as a result legal restrictions have been levied on its collection from the wild (Kumar et al., 2017). The scarcity of herbal raw material not only caused economic constraints on local communities but also resulted in adulteration of *P. kurroa*, thereby affecting quality and efficacy of drugs due to lower levels of picroside-I (P-I) and picroside-II (P-II), the signature bioactive compounds of *P. kurroa* (Shitiz et al., 2013).

The picrosides, P-I and P-II, possess vast therapeutic potential and their bio-efficacy has also been tested *via* both *in vitro* as well as *in vivo* studies (Kumar et al., 2017, **Table 1**). Moreover, the herbal drug formulations required both P-I and P-II in a ratio of 1:1.5 (Kumar et al., 2017). Therefore, to provide a constant supply of raw material without sacrificing the levels of major chemical constituents, mass propagation of quality plant material is a pressing need. Mass propagation of *P. kurroa* shoots through *in vitro* culture has been optimized but with lower yields of P-I (Sood and Chauhan, 2010). Moreover, the content of P-I is also ∼5-fold lesser in *P. kurroa* shoots grown *in vitro* compared to those available in natural habitats (Sood and Chauhan, 2010; Shitiz et al., 2015; Sharma et al., 2016).

**Table 1:** Bioactivities of P-I and P-II.

Iridoid glycosides	Treatments	Reference
P-II	Hind limb ischemia reperfusion injury	Comu et al., 2016
	Anti-allergic asthma	Choi et al., 2016
	Renal ischemia/reperfusion injury	Wang L. et al., 2015
	Neuroprotective	Guo et al., 2010; Li et al., 2010; Pei et al., 2012; Liu et al., 2014; Zhao et al., 2014; Wang T. et al., 2015
	Hepatoprotective	Gao and Zhou, 2005a,b
	Hypoxia/reoxygenation induced cardiomyocyte injury	Meng et al., 2012a,b
	Myocardial ischemia reperfusion injury	Wu et al., 2014
P-I	Hepatoprotective	Dwivedi et al., 1992
	Anti-cancer	Rathee et al., 2013
P-I and P-II	Neuritogenesis	Li et al., 2000, 2002

In *P. kurroa*, it has been hypothesized that the P-I and P-II are biosynthesized in a tissue specific manner, i.e., shoots synthesize P-I, roots contained only P-II and stolons/rhizomes produce both P-I and P-II (Pandit et al., 2013a). The shoots of *P. kurroa* have been established as biosynthetic tissues of only P-I as P-II was not detected in *P. kurroa* shoots and the P-I content also showed progressive increase in different growth and developmental stages of *P. kurroa* shoots (Sood and Chauhan, 2010; Pandit et al., 2013a; Kumar et al., 2015b). In contrast, both P-I and P-II contents have been detected and showed progressive increase in different growth and developmental stages of *P. kurroa* stolons/rhizomes. In previous reports, we had suggested that rhizomes are a storage tissue for P-I and P-II not the biosynthetic tissue, whereas P-I was postulated as biosynthesized in shoots and possibly transported to rhizomes (Sood and Chauhan, 2010; Pandit et al., 2013a). The root tissues of *P. kurroa* contained only P-II which was detected at the later stage compared to rhizome and thus, it was hypothesized that P-II might be transported from rhizomes to roots (Pandit et al., 2013a). Therefore, it would be of paramount importance if P-II can be produced in shoots, which will not only relieve pressure from natural habitat of *P. kurroa* for uprooting rhizomes/roots but will also provide a continuous supply of herbal raw material through shoot cultures with desired amounts of P-I and P-II. However, lack of knowledge on P-II biosynthesis has precluded implementing any genetic intervention strategy toward enriching P-II in *P. kurroa* shoot cultures.

**FIGURE 1:**
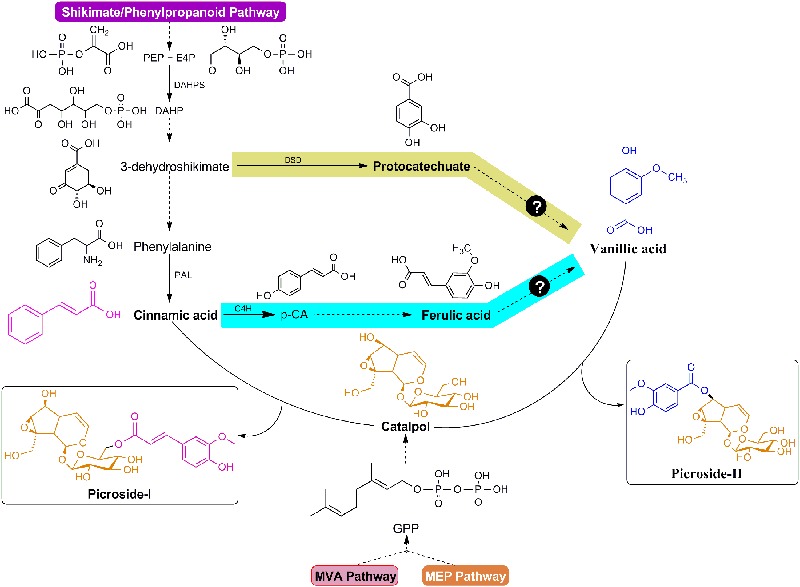
**Modular design of P-I and P-II biosynthetic pathways.** The structures of P-I and P-II linked to cinnamic acid/vanillic acid (purple/blue color) moieties and catalpol (orange color). Question marks indicate plausible routes for vanillic acid production.

In previous studies, maximum efforts have been focused on P-I production in *P. kurroa* as P-II was not produced under *in vitro* shoot cultures. The biosynthetic pathways for P-I and P-II were, therefore, proposed including; mevalonate (MVA), non-mevalonate (MEP), shikimate/phenylpropanoid, and iridoid pathways so as to pinpoint key genetic intervention points to maximize picrosides content using metabolic engineering approaches (Kumar et al., 2013; Shitiz et al., 2015). It has been hypothesized that the catalpol (CAT) backbone derived from iridoid pathway undergoes esterification reactions with *trans*-cinnamic acid (CA) and vanillic acid (VA) to produce P-I and P-II, respectively (Kumar et al., 2013). The metabolic route of P-I biosynthesis has been ascertained through precursor feeding and enzyme inhibitor studies along with the assortment of key candidate genes through expression analysis using qRT-PCR vis-à-vis P-I content (Pandit et al., 2013b; Kumar et al., 2015b, 2016b,c; Shitiz et al., 2015). However, the complexity of P-II biosynthesis is still unresolved as both protocatechuate and ferulic acid (FA); the metabolites of shikimate/phenylpropanoid pathway, are plausible to produce VA, the immediate precursor of P-II (**Figure 1**) (Kawoosa et al., 2010; Gahlan et al., 2012; Kumar et al., 2013). In this study, we have selected mature stolons to elucidate the mechanism controlling P-II biosynthesis in *P. kurroa*.

To elucidate the metabolic fluxes, radioactive/stable isotopes labeled flux analysis has been used in various plant species (Ishihara et al., 2015; Xiong et al., 2015) but concurrently accompanied with cost intensive and time consuming experiments (Stephanopoulos, 2012). Therefore, in this study, we have utilized natural variation in P-II content among different accessions (genotypes) of *P. kurroa* so as to guide us in discerning the route and flux of intermediates leading to the biosynthesis of P-II. Moreover, the integration of metabolites contents data with the corresponding transcriptomes would enable us to understand the flux of intermediates vis-à-vis gene transcripts controlling P-II biosynthesis in *P. kurroa*.

## Materials and Methods

### Collection of Plant Material

A total of 10 accessions of 1 year old *P. kurroa* were collected from different locations of North-Western Himalaya, India. The details of accessions are provided in Supplementary Table 1. All the accessions were kept in the greenhouse of Jaypee University of Information Technology, Waknaghat, Himachal Pradesh, India (31°0′58.55″ N and 77°4′12.63″ E; 1700 m, altitude). The plants were washed with tap water and segregated into shoots, mature stolons, and roots. The shoots were coded with PKS, while mature stolons were labeled as PKST. In contrast, roots were labeled as PKR. We have used PKS and PKST tissues in this study. All tissue samples were kept at -80°C for further analyses.

### Extraction and Quantification of P-I and P-II Contents

The P-I and P-II were extracted in triplicates from shoots and stolons tissues of all 10 *P. kurroa* accessions as per the protocol described in Kumar et al. (2016c). The quantification of P-I and P-II in all the samples was performed in triplicates by using HPLC method as described in Pandit et al. (2013a).

### Extraction and Quantification of Intermediate Metabolites

Four accessions with variation for P-I content in shoots (PKS-1, PKS-4, PKS-5, and PKS-21) and with variation for P-I and P-II contents in stolons (PKST-3, PKST-5, PKST-16, and PKST-18) were subjected to the extraction of intermediate metabolites viz. CA, *p*-coumaric acid (*p*-CA), FA, protocatechuic acid (PA), and VA. The extraction was performed as per the method described in Mattila and Kumpulainen (2002) with some modifications. The samples (200 mg) were weighed and homogenized with liquid N_2_ in prechilled pestle and mortar. To each homogenized sample, 7 mL of combination of methanol [containing 2 g/L butylated hydroxytoluene (BHT)] and 10% acetic acid in a ratio of 85:15 was added and vortexed for 10 min at room temperature (25°C). The volume of extracts was made up to 10 mL with distilled water and mixed properly. Each extract was then transferred to 100 mL reagent bottle and suspended with 12 mL of distilled water and 5 mL 10 M sodium hydroxide. The contents of the extracts were then bubbled with N_2_ gas, sealed and kept on a magnetic stirrer for overnight at room temperature (25°C). On following day, the pH of extracts was adjusted to 2 with concentrated hydrochloric acid. The metabolites released in the solution were then extracted three times with 15 mL of a mixture of cold diethyl ether and ethyl acetate in a ratio of 1:1 by shaking and centrifuging for 10 min at 7000 rpm and 4°C temperature condition. The diethyl ether/ethyl acetate layers thus obtained, were evaporated to dryness and reconstituted in 1.5 mL of absolute methanol. The obtained samples were filtered through 0.22 μm filter (Millipore) and subjected to quantification of selected metabolites by using HPLC as per the method described in Nour et al. (2013). The selected compounds viz. CA, *p*-CA, and FA were detected at a wavelength of 290 ± 4 nm, 310 ± 4 nm, and 330 ± 4 nm, respectively while PA and VA were detected at an absorbance of 260 ± 4 nm. All the selected compounds were identified upon comparison of retention time and UV spectra with standards (Sigma–Aldrich, USA). The extraction and quantification experiments were performed in triplicates.

### Feeding of *In vitro* Grown *P. kurroa* Shoots with Different Precursors

The solutions of different precursors including, *p*-CA, PA, and FA (Sigma-Aldrich, USA) were prepared as neutral aqueous stocks at 150 μM concentrations. Conversely, 150 μM aqueous solution of VA (Sigma-Aldrich, USA) was mixed with 70 μM CAT (Sigma-Aldrich, USA) and used for the feeding of *in vitro* grown *P. kurroa* shoots. These concentrations were selected based on their effects on P-I production in *P. kurroa* when applied alone and in combination under tissue culture conditions (Kumar et al., 2016b). Following this, the solutions of different precursors were added into the culture tubes containing optimized MS medium [containing 3 mg/L indole-3-butyric acid (IBA) and 1 mg/L kinetin (KN)] after filter sterilization through 0.22 μm syringe filter (Millipore) (Sharma et al., 2015). In each culture flask, the shoot explant of *P. kurroa* grown at 25 ± 2°C (having negligible P-I content) was aseptically transferred and all the cultures were kept in a plant tissue culture chamber maintained at 15 ± 2°C (temperature favors P-I production) (Sood and Chauhan, 2010). The *P. kurroa* shoots without precursor’s treatment were referred to as controls. The experiment was performed in triplicates. The shoot samples were collected after 30 days and immediately stored at -80°C for further analyses. All the samples were subjected to the extraction and quantification of CA, *p*-CA, FA, VA, P-I, and P-II metabolites in triplicates as per the protocol mentioned above.

### Culturing of *P. kurroa* Shoots Grown *In vitro* with Different Concentrations of VA+CAT Mixture and P-II

The different concentrations of VA+CAT mixture viz. 25 μM each VA+CAT, 70 μM each VA+CAT, 70 μM VA with 150 μM CAT, 150 μM each VA+CAT and 230 μM each VA+CAT, were also tested after determination of P-II content in shoot cultures fed with different precursors. Moreover, P-II at a concentration of 25 μM was also introduced separately in the MS medium to check the accumulation of P-II in *P. kurroa* shoots. The culturing of *P. kurroa* shoots was performed according to the same protocol as mentioned above. The untreated shoots were labeled as controls. The experiment was performed in triplicates. The shoot samples were harvested after 30 days and immediately stored at -80°C for the analysis of P-I and P-II contents. The analysis of picrosides was carried out in triplicates.

### Treatment of *In vitro* Grown *P. kurroa* Shoots with VA+CAT Mixture and P-II under Liquid Culture Conditions

The solutions of VA+CAT mixture and P-II were prepared at concentrations of 70 and 25 μM, respectively to check the intake of precursors by the plant. These solutions were then incorporated into the culture tubes having optimized liquid MS medium (containing 3 mg/L IBA and 1 mg/L KN) after filter sterilization through 0.22 μm syringe filter (Millipore). The culturing of *P. kurroa* shoots was performed under the same conditions as mentioned above. The untreated shoots were labeled as controls. The experiment was performed in triplicates. The shoot samples were harvested after 30 days and immediately stored at -80°C for the further analyses. The analysis of VA and P-II contents was performed in both shoots and liquid media left after the harvesting and carried out in triplicates.

### Isolation of Total RNA, Preparation of Libraries, and Sequencing for RNA-seq Analysis

Total RNA was isolated from four accessions of shoots (PKS-1, PKS-4, PKS-5, and PKS-21) and stolons (PKST-3, PKST-5, PKST-16, and PKST-18) by using total RNA isolation kit (RaFlex^TM^) according to the instructions mentioned in the user manual. The construction of libraries and their sequencing for transcriptome analysis was performed as per the protocol described in Pal et al. (2015).

### Transcriptome *De novo* Assembly and Validation

For *de novo* assembly, we chose go with De brijn graph based Trinity Assembler based on the criteria of; (a) default K-mer, (b) less memory foot print, (c) optimized for Illumina paired end data, (d) reproducibility, and (e) configurable for all computing capacities (Henschel et al., 2012). To pool assembly, 50 core threads of processing with 2.4 GHz speed and a maximum Heap Space of 50 GB was allotted. Nevertheless, *de novo* transcriptome assemblers are capable of producing in fragmented/miss-assembly, the validation of assembled transcriptome was carried out by mapping back the high quality (HQ) filtered reads to the ESTs. The HQ filtered reads from each library thus obtained were mapped to the assembled transcriptome by using Bowtie software. This analysis generated.bam (Binary Sequence Alignment/Map) files which were processed through bedtools and samtools for quantitation (read count estimation) of each transcript in a library and also to calculate the total coverage and average depth of the transcriptome in each library (Li et al., 2009; Quinlan and Hall, 2010).

### Transcripts Annotation

The annotation and statistical analysis of each transcript was performed by homology based method against NCBI nrdb protein database using Blast2GO software through Blastx module (Conesa et al., 2005). The parameters used for this analysis were; (a) e-Value b = 10-e4, (b) Similarity ≥35%, (c) Annotation cutoff ≥55, (d) GO weight cutoff ≥5. Moreover, domain level annotation was also performed by using the Online InterProScan tool RunIprScan-1.1.0^1^ (Henschel et al., 2012).

### Normalization of Transcripts and their Expression Profiling

The benefit of using NGS based transcriptome profiling is to recognize sample/condition specific expressed transcripts which was not easy with earlier hybridization methods. Transcripts with a read count of ≥10 in any one of the libraries were considered to be expressed. To perform normalization and expression profiling, a sub bam file was created from the master bam file using RSEM software (Li and Dewey, 2011). In RSEM, the default parameters/commands were used to normalize each library which provides an output with expected normalized read count, TPM (tags per million) and FPKM (fragments per kilobase per million). Log to the base 2 of FPKM was considered as absolute expression or Delta CT equivalent value.

### Transcripts Mapping, Differentially Expressed Gene (DEG), and Correlation Analysis

The annotated transcripts obtained from BLAST2GO were mapped to Kyoto Encyclopedia of Genes and Genomes (KEGG) (Kanehisa et al., 2016). Transcripts which were involved in secondary metabolic pathways were shortlisted for DEG analysis using sequence similarity threshold of >98%. Only those transcripts were selected and used for the analyses which were present in all the eight different samples, i.e., four accessions of each shoots and stolons. Hierarchical clustering of selected DEGs were performed using heatmaply, gplot2, plotly packages in R. Further, the correlation analysis was carried out by using R-package through “Corrgram” function (Friendly, 2002).

### Preparation of Complementary DNA (cDNA) and Expression Analysis of Selected Genes through Quantitative Real Time Polymerase Chain Reaction (qRT-PCR)

The cDNA of four different shoots (PKS-1, PKS-4, PKS-5, and PKS-21) and stolons accessions (PKST-3, PKST-5, PKST-16, and PKST-18) was synthesized from 5 μg of total RNA by using Verso cDNA synthesis kit (Thermo Scientific, USA) according to the manufacturer’s instructions. The cDNA of each sample thus obtained was quantified by using ND-2000 UV spectrophotometer and diluted with nuclease free water to obtain 100 ng concentration. For gene expression analysis, six genes were selected and subjected to qRT-PCR analysis. The primers of the selected genes were designed from transcriptomic sequences of *P. kurroa* through Primer3 software and the details with annealing temperature are provided in Supplementary Table 2. The gene expression analysis was performed in triplicates according to the method described in Kumar et al. (2015b). The housekeeping gene, 26S was used as reference in this study for normalization of gene expression.

### Statistical Analyses

The one and two-way analysis of variance (ANNOVA) followed by a Bonferroni test were performed from data in triplicates (mean ± SD) using GraphPad prism software version 6.0. To examine the correlations between gene expression profiles obtained from RNA-seq and qRT-pCR, log2 fold change values were estimated among different shoots and stolons accessions. The scatterplots were then created by comparing log2 fold change values determined by RNA-seq and qRT-PCR using GraphPad prism software version 6.0. The correlations were represented in terms of coefficient of determination, i.e., R^2^.

## Results

### Picrosides (P-I and P-II) Content in Different Accessions of *P. kurroa*

**FIGURE 2:**
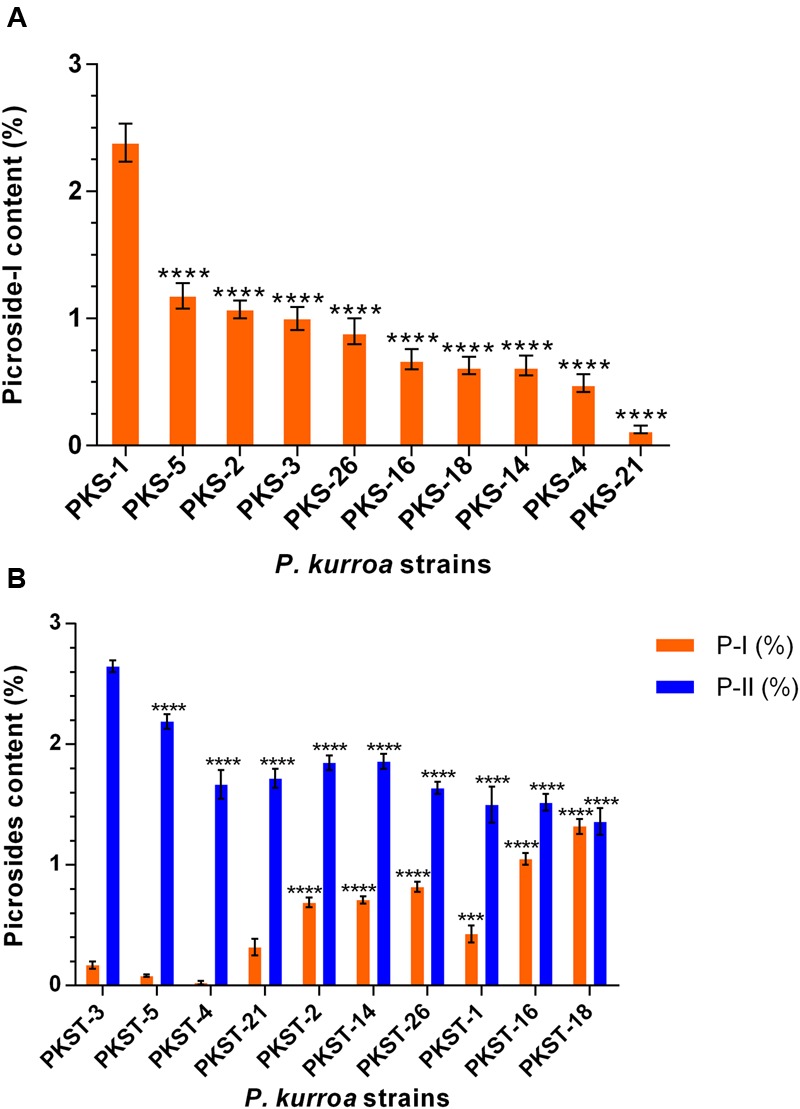
**Determination of picrosides contents in different tissues of *Picrorhiza kurroa* accessions; (A)** shoots and **(B)** stolons. The data presented as means ± SD (*n* = 3). Significance was evaluated within picrosides contents between different accessions (^∗∗∗^*p* < 0.001, ^∗∗∗∗^*p* < 0.0001).

A total of 10 different *P. kurroa* accessions were analyzed for P-I and P-II contents separately in shoots and stolons compartments. The analysis of shoot tissues of different *P. kurroa* accessions revealed significant variations in P-I content (**Figure 2A**). The maximum level of P-I was observed in PKS-1, i.e., 2.38% which was significantly higher (*p* < 0.0001) as compared to PKS-5 (1.18%), PKS-2 (1.07%), PKS-3 (1%), PKS-26 (0.9%), PKS-16 (0.68%), PKS-18 (0.63%), PKS-14 (0.63%), PKS-4 (0.49%), and PKS-21 (0.13%), respectively (**Figure 2A**). In contrast, P-II was not detected in shoots of *P. kurroa* plants. It was evident from the results that PKS-1 and PKS-5 showed maximum P-I content and thus, referred to as high P-I content accessions of *P. kurroa*. In contrast, PKS-4 and PKS-21 showed minimum P-I content and thus, referred to as low P-I content accessions of *P. kurroa*.

Upon analysis of stolons portions of different *P. kurroa* accessions, significant modulations in both P-I and P-II contents were observed (**Figure 2B**). The maximum P-II content, i.e., 2.65% was observed in PKST-3 which showed significant progressive decrease leading to PKST-18 with 1.36% P-II content (*p* < 0.0001) (**Figure 2B**). In contrast, the minimum P-I content of 0.08% was detected in PKST-5 followed by non-significant increase in PKST-3 stolon (0.17%). Further, the stolons of PKST-1, PKST-2, PKST-14, PKST-26, PKST-16, and PKST-18 showed significant progressive increase with 0.43% (*p* < 0.001), 0.69% (*p* < 0.0001), 0.71% (*p* < 0.0001), 0.82% (*p* < 0.0001), 1.05% (*p* < 0.0001), and 1.32% (*p* < 0.0001), respectively P-I content compared to PKST-5 (**Figure 2B**). These observations implies that PKST-3 and PKST-5 stolons contained maximum P-II content but minimum P-I content and thus, referred to as high P-II content accessions of *P. kurroa*. Conversely, PKST-16 and PKST-18 stolons contained minimum P-II but maximum P-I content and thus, referred to as low P-II content accessions of *P. kurroa*.

### Alterations in the Levels of Intermediate Metabolites of Shikimate/Phenylpropanoid Pathway among Selected *P. kurroa* Accessions

**FIGURE 3:**
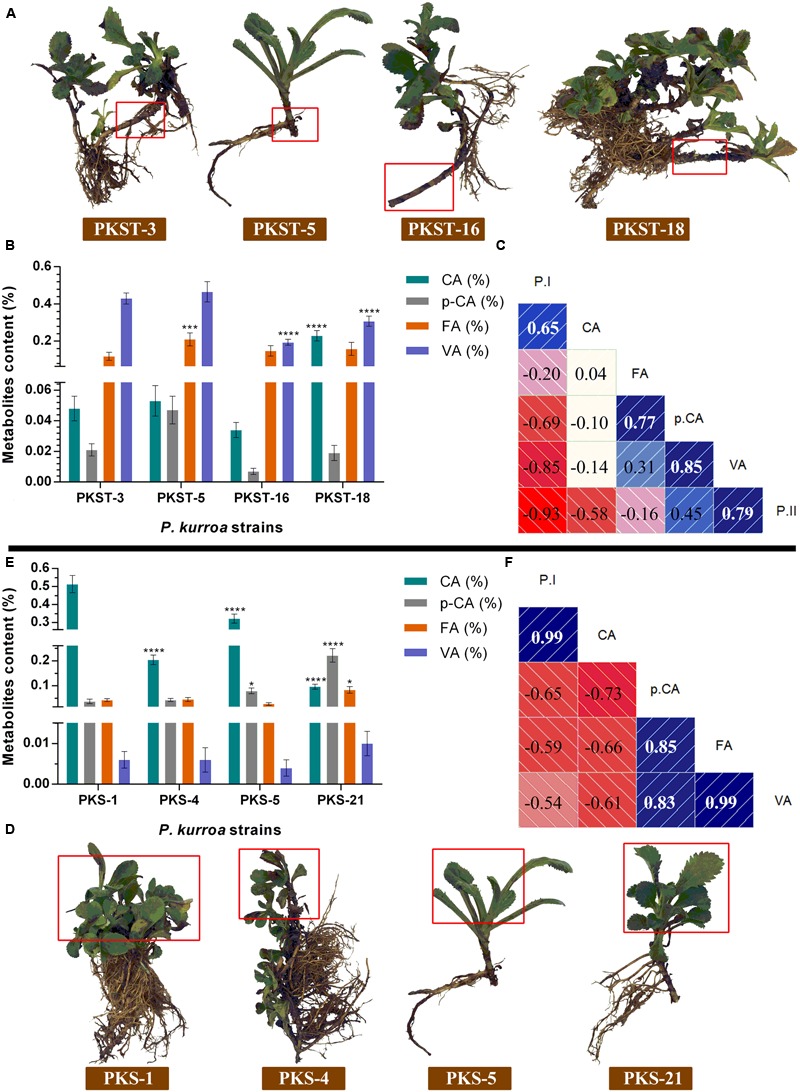
**Comparative analysis of intermediate metabolites contents among different tissues of *P. kurroa* accessions; (A,D)** different *P. kurroa* accessions selected for stolons and shoots, respectively; **(B,E)** variations in metabolites contents between stolons and shoots, respectively and; **(C,F)** correlogram showing correlations between tested metabolites and picrosides contents among stolons and shoots, respectively. Correlations are presented in the form of graphs filled in proportion to the Pearson’s correlation coefficient values. Clock-wise occupied with blue color depict positive correlations while anti-clockwise graphs filled with red color indicate negative correlations. The data presented as means ± SD (*n* = 3). Significance was evaluated within metabolites contents between different accessions (^∗^*p* < 0.05, ^∗∗∗^*p* < 0.001, ^∗∗∗∗^*p* < 0.0001).

The shoots and stolons of *P. kurroa* accessions selected for high and low P-I/P-II contents were examined for the levels of intermediate metabolites in shikimate/phenylpropanoid pathway viz. CA, *p*-CA, FA, PA, and VA (**Figures 3A–F**). The investigation of shoot tissues revealed that PKS-1 contained maximum CA content of 0.5% which showed significant progressive decrease in PKS-5, PKS-4, and PKS-21 with 1.5- (*p* < 0.0001), 2.5- (*p* < 0.0001), and 5.3-fold (*p* < 0.0001), respectively (**Figure 3E**). These results are akin to P-I accumulation patterns in selected shoot tissues of *P. kurroa*. In contrast, the lowest *p*-CA content (0.04%) was observed in PKS-1 and showed significant increase in PKS-5 and PKS-21 with 2.1- (*p* < 0.05) and 5.9-fold (*p* < 0.0001), respectively. The *p*-CA content of PKS-4 showed non-significant modulation compared to PKS-1 shoots (**Figure 3E**). The examination of FA content in shoot tissues revealed 2-fold (*p* < 0.05) significant elevation in PKS-21 compared to PKS-1, PKS-5, and PKS-4 accessions (**Figure 3E**). Moreover, the VA content showed non-significant modulation among all the four selected shoots of *P. kurroa*. The PA content was not detected in the selected shoots of *P. kurroa*. It was evident from these results that CA is positively correlated with P-I content in shoots with Pearson correlation coefficient (PCC) of 0.99 (**Figure 3F**).

The investigation of four different stolons revealed that PKST-18 contained maximum CA content of 0.2% which showed significant increase with 4.7-fold (*p* < 0.0001) compared to PKST-3 (**Figure 3B**). The CA content of PKST-3 showed non-significant modulation with PKST-5 and PKST-16 stolons. The analysis of *p*-CA content showed non-significant alterations in all the four selected stolons of *P. kurroa*. Upon examination of FA content, we observed highest level in PKST-5 (0.2%) which exhibited 1.7-fold (*p* < 0.001) significant increase compared to PKST-3, PKST-16, and PKST-18 (**Figure 3B**). In contrast, highest VA content was observed in PKST-3 and PKST-5 followed by significant decrease in PKST-16 and PKST-18 with 2.2- (*p* < 0.0001) and 1.3-fold (*p* < 0.0001), respectively (**Figure 3B**). Moreover, PA content was not observed in the stolons of *P. kurroa*. These observations infer positive correlations of *p*-CA and VA with P-II through PCC of 0.45 and 0.79, respectively (**Figure 3C**).

### Effect of Treatment with Different Precursors on Picrosides and Intermediate Metabolites Contents

**FIGURE 4:**
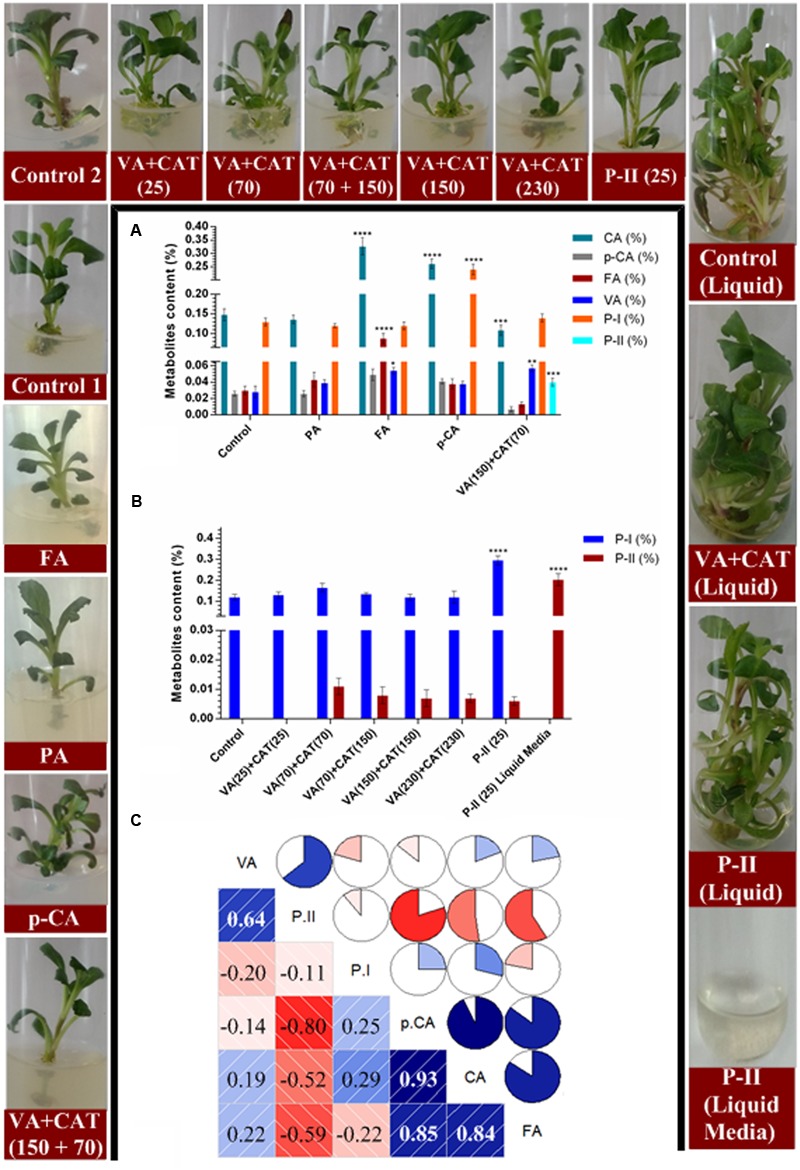
**Effects of various precursor’s feeding on the contents of tested metabolites; (A)** variations in metabolites contents between different precursor treatments, i.e., PRCA, FA, and *p*-CA at 150 μM concentration along with 150 μM VA + 70 μM CAT; **(B)** variations in picrosides contents among different concentrations of VA+CAT along with P-II and liquid media remained after the collection of samples and; **(C)** correlogram showing correlations between tested metabolites and picrosides contents among different precursors treated samples. Correlations are presented in the form of pie graphs filled in proportion to the Pearson’s correlation coefficient values. Clock-wise occupied with blue color depict positive correlations while anti-clockwise pie graphs filled with red color indicate negative correlations. The data presented as means ± SD (*n* = 3). Significance was evaluated within metabolites contents between different precursor’s treatments (^∗^*p* < 0.05, ^∗∗^*p* < 0.01, ^∗∗∗^*p* < 0.001, ^∗∗∗∗^*p* < 0.0001).

The *in vitro* grown shoots of *P. kurroa* were treated with 150 μM concentration of PA, FA, and *p*-CA along with combination of 150 μM VA and 70 μM CAT to observe their effects on the CA, *p*-CA, FA, VA, P-I, and P-II contents (**Figures 4A–C**). The analysis of CA content revealed 2.2- (*p* < 0.0001) and 1.7-fold (*p* < 0.0001) significant increase in shoots treated with FA and *p*-CA, respectively, whereas 1.3-fold (*p* < 0.001) significant decrease was observed in VA + CAT fed shoots compared to *P. kurroa* shoots without treatment, i.e., control shoots (**Figure 4A**). The *p*-CA content, on the other hand showed non-significant modulations in all the fed shoots compared to control. The investigation of FA content revealed significant increase with 2.9-fold (*p* < 0.0001) in shoots treated with FA compared to untreated shoots. In contrast, 1.9- (*p* < 0.05) and 2-fold (*p* < 0.01) significant elevation in VA content was observed in shoots fed with FA and VA + CAT, respectively compared to control (**Figure 4A**). Moreover, the examination of P-I content revealed 1.8-fold (*p* < 0.0001) significant enhancement in shoots fed with *p*-CA compared to control. Conversely, small level of P-II, i.e., 0.04% was only detected in *P. kurroa* shoots treated with VA + CAT (**Figure 4A**). Thus, it was evident from these results that VA and P-II shared high positive correlation with PCC of 0.64 and the former was also positively correlated with CA and FA with PCC of 0.19 and 0.22, respectively (**Figure 4C**).

Since, P-II was only detected in VA + CAT fed shoots therefore, different concentrations of VA + CAT were further tested to observe their influence on the P-II content. The data revealed non-significant alterations in P-II content among *P. kurroa* shoots treated with different concentrations of VA + CAT (**Figure 4B**). Moreover, the P-II content also exhibited non-significant alteration in shoots fed with P-II while 0.2% P-II was observed in the media remained after the sampling of shoots treated with P-II (**Figure 4B**).

The feeding of different precursors *in vitro* also increased shoot biomass compared to untreated control which might be due to the enhanced carbon source utilization.

### Correlation Analysis between Picrosides Content and Transcripts Abundance Values (FPKM)

To get insight into flux dynamics of different metabolic modules connected to picrosides biosynthesis, correlation maps were constructed between the FPKM values of selected transcripts and picrosides content among different shoots and stolons accessions of *P. kurroa*^2^. The transcripts encoding enzymes catalyzing regulatory reactions in different metabolic pathways were selected based on their connection with picrosides biosynthesis. The details of selected transcripts with their role in picrosides production are provided in **Table 2**.

**Table 2:** Details of transcripts encoding enzymes selected for this study and their plausible involvement in picrosides biosynthesis.

Transcript encoding enzymes	Abbreviations	Function/pathway	Flux to picrosides [Convergent (C)/ Divergent (D)]		Reference
			P-I	P-II	
1-Deoxy-D-xylulose-5-phosphate synthase	DXPS	IPP and DMAPP production	C	C	Kumar et al., 2015b
3-Deoxy-D-arabinoheptulosonate 7-phosphate synthase	DAHPS	Entry step of Shikimate pathway	C	C	Kumar et al., 2016b
Hydroxymethylglutaryl-CoA reductase	HMGR	IPP production	C	C	Kumar et al., 2015b
3-Hydroxyacyl CoA dehydrogenase	HADH	Benzoic acid biosynthesis	D	D	Widhalm and Dudareva, 2015
Anthranilate synthase	AS	Tryptophan biosynthesis	D	D	Galili et al., 2016
Tyrosine decarboxylase	TDC	Tyrosine degradation	D	D	Yamada and Sato, 2016
Caffeic acid-3-*O*-methyltransferase	CMT	Ferulic acid production	D	C	Trabucco et al., 2013
Chalcone synthase	CHS	Flavonoids biosynthesis	D	D	Zuk et al., 2016
Chorismate mutase	CM	Phenylalanine biosynthesis	C	C	Kumar et al., 2016b
Cinnamic acid-4-hydroxylase	C4H	*p*-Coumaric acid production	D	C	Kumar et al., 2016b
Cinnamoyl CoA reductase	CCR	Guaiacyl lignin biosynthesis	D	D	Humphreys and Chapple, 2002
Ferulic acid-5-hydroxylase	F5H	Syringyl lignin biosynthesis	D	D	Wagner et al., 2015
Farnesyl pyrophosphate synthase	FPPS	Sesquiterpene biosynthesis	D	D	Sun et al., 2016
Geraniol synthase	GS	Monoterpene biosynthesis	C	C	Kumar et al., 2015b
Geranyl pyrophosphate synthase	GPPS	Terpene biosynthesis	C	C	Shitiz et al., 2015
Geranylgeranyl pyrophosphate synthase	GGPPS	Diterpene biosynthesis	D	D	Kumar et al., 2016a
Glucose-6-phosphate dehydrogenase	G6PDH	Pentose phosphate pathway	C	C	Kumar et al., 2015b
Hexokinase	HK	Glycolysis	C	C	Kumar et al., 2016c
Isocitrate dehydrogenase	ICDH	TCA cycle	D	D	Kumar et al., 2016c
Pytoene synthase	PYS	Carotenoid biosynthesis	D	D	Simpson et al., 2016
Pyruvate kinase	PK	Glycolysis	C	C	Kumar et al., 2016c
Squalene synthase	SQS	Triterpene biosynthesis	D	D	Sun et al., 2016
4-Coumarate CoA ligase	4CL	Flavonoids biosynthesis	D	D	Humphreys and Chapple, 2002
Phenylalanine ammonia lyase	PAL	Cinnamic acid production	C	C	Kumar et al., 2016b
Phosphoenolpyruvate carboxykinase	PEPCK	Phosphoenolpyruvate production	C	C	Delgado-Alvarado et al., 2007

**FIGURE 5:**
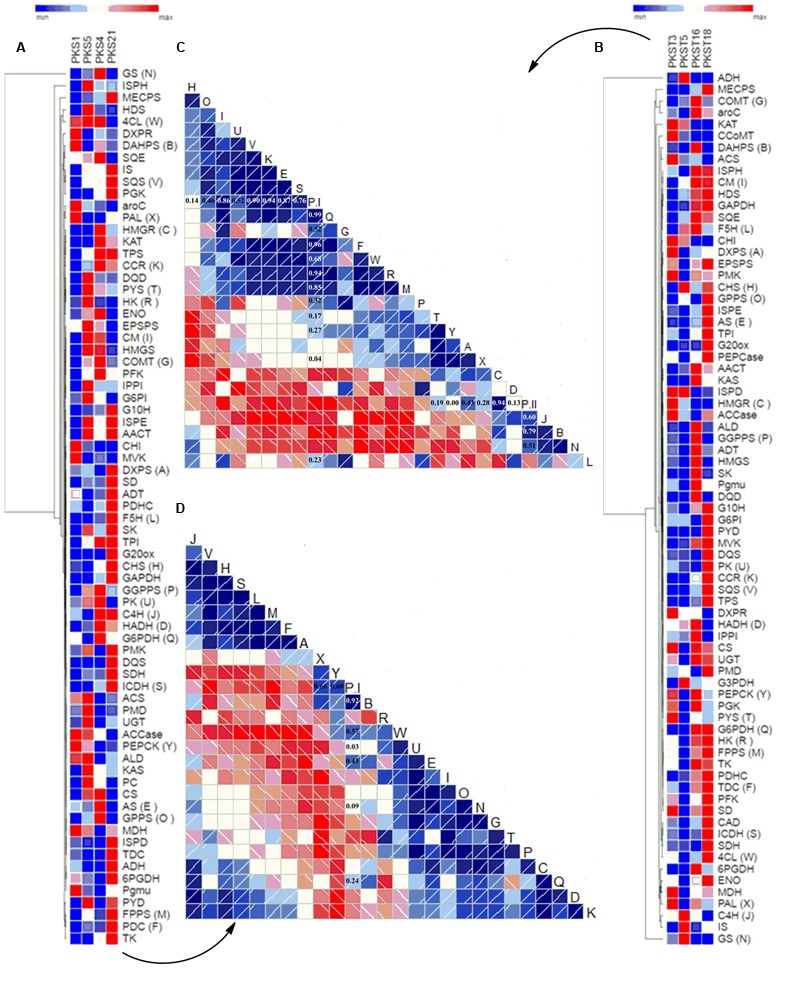
**Differential expression analysis of genes involved in secondary metabolism among different tissues of *P. kurroa* accessions; (A,B)** cluster analysis of differentially expressed genes patterns between different *P. kurroa* accessions selected for shoots and stolons, respectively; **(C,D)** correlations in terms of Pearson’s correlation coefficients depicted using correlogram between selected genes and metabolites contents among different stolons and shoots, respectively. The data is represented in the form of graphs filled in proportion to the Pearson’s correlation coefficient values. Clock-wise occupied with blue color depict positive correlations while anti-clockwise graphs filled with red color indicate negative correlations.

The correlation analysis among selected accessions of shoots viz. PKS-1, PKS-5, PKS-4, and PKS-21 revealed positive correlations of *PAL* (0.66), *PEPCK* (0.60), *DAHPS* (0.92), *4CL* (0.57), *PK* (0.03), *AS* (0.45), *GS* (0.09), and *G6PDH* (0.24) with P-I content while rest of the selected transcripts showed negative correlations (**Figures 5A,D**). In contrast, the correlation analysis among selected accessions of stolons viz. PKST-3, PKST-5, PKST-16, and PKST-18 revealed positive correlations of *PYS* (0.19), *DXPS* (0.43), *PAL* (0.28), *HMGR* (0.94), *HADH* (0.13), *C4H* (0.60), *DAHPS* (0.79), and *GS* (0.51) with P-II content while remaining selected transcripts showed negative correlations (**Figures 5B,C**). These observations implied that *DAHPS, PAL, PEPCK*, and *4CL* which showed high positive correlations (>0.60) with P-I content might be considered as the key candidate genes for P-I biosynthesis. Conversely, *HMGR, C4H, DAHPS*, and *GS* might play an important role in the biosynthesis of P-II in *P. kurroa*.

### Gene Expression Profiling among Selected Accessions of *P. kurroa*

**FIGURE 6:**
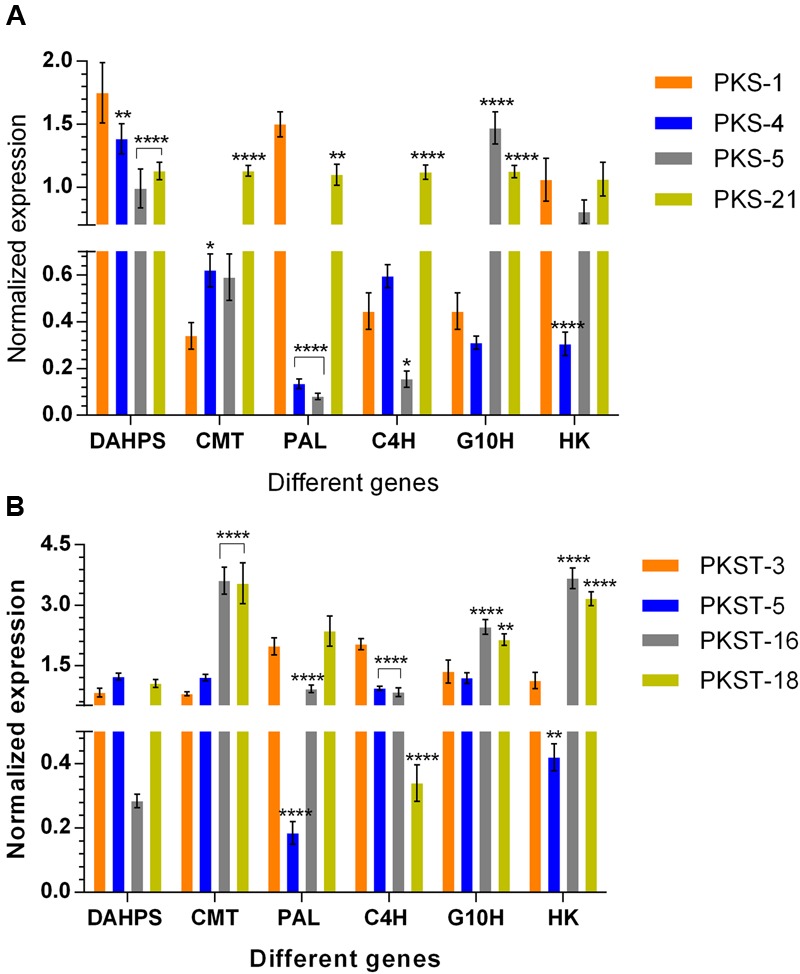
**Expression profiling of selected genes in different tissues of *P. kurroa* accessions; (A)** shoots and **(B)** stolons. Expression values were normalized with levels of 26S reference gene. The data presented as means ± SD (*n* = 3). Significance was evaluated for each gene between different accessions (^∗^*p* < 0.05, ^∗∗^*p* < 0.01, ^∗∗∗∗^*p* < 0.0001).

To confirm the differential gene expression patterns obtained through transcriptomes analysis, six genes were randomly selected (having both positive and negative correlations with P-I and P-II contents) and further tested using qRT-PCR. Among the selected different accessions of shoots, the transcripts levels of *DAHPS* showed 1.3- (*p* < 0.01), 1.8- (*p* < 0.0001), and 1.6-fold (*p* < 0.0001) significant decrease in PKS-4, PKS-5, and PKS-21, respectively compared to PKS-1 (**Figure 6A**). The expression level of *CMT* displayed 1.8- (*p* < 0.05) and 3.3-fold (*p* < 0.0001) significant increase in PKS-4 and PKS-21, respectively compared to PKS-1 (**Figure 6A**). In contrast, the expression level of *PAL* was maximum in PKS-1 which showed significant decrease in PKS-4, PKS-5, and PKS-21 with 10.7- (*p* < 0.0001), 18- (*p* < 0.0001), and 1.3-fold (*p* < 0.01), respectively (**Figure 6A**). The transcript levels of *C4H* exhibited significant elevation in PKS-21 with 2.4-fold (*p* < 0.0001), while 2.8-fold (*p* < 0.05) significant decrease was observed in PKS-5 compared to PKS-1 (**Figure 6A**). Moreover, the transcript level of *G10H* showed 3.3- (*p* < 0.0001) and 2.5-fold (*p* < 0.0001) significant increment in PKS-5 and PKS-21, respectively compared to PKS-1 (**Figure 6A**). The expression level of *HK* showed 3.4-fold (*p* < 0.0001) significant decrease in PKS-4 compared to PKS-1 (**Figure 6A**).

The expression analysis of *DAHPS* gene among different accessions of stolons showed non-significant modulations (**Figure 6B**). The expression level of *CMT* displayed 4.5- (*p* < 0.0001) and 4.4-fold (*p* < 0.0001) significant increase in PKST-16 and PKST-18, respectively compared to PKST-3 (**Figure 6B**). In contrast, the expression level of *PAL* showed 10.4- (*p* < 0.0001) and 2.2-fold (*p* < 0.0001) significant decrease in PKST-5 and PKST-16, respectively compared to PKST-3 (**Figure 6B**). The transcript level of *C4H* was maximum in PKST-3 which showed significant decrease in PKST-5, PKST-16, and PKST-18 with 2.1- (*p* < 0.0001), 2.4- (*p* < 0.0001), and 5.9-fold (*p* < 0.0001), respectively (**Figure 6B**). The transcript level of *G10H* exhibited 1.8- (*p* < 0.0001) and 1.6-fold (*p* < 0.01) significant increment in PKST-16 and PKST-18, respectively compared to PKST-3 (**Figure 6B**). Moreover, the expression level of *HK* showed 3.2- (*p* < 0.0001) and 2.8-fold (*p* < 0.0001) significant elevation in PKST-16 and PKST-18, respectively, while 2.6-fold (*p* < 0.01) significant decrease in PKST-5 was observed compared to PKST-3 (**Figure 6B**).

**FIGURE 7:**
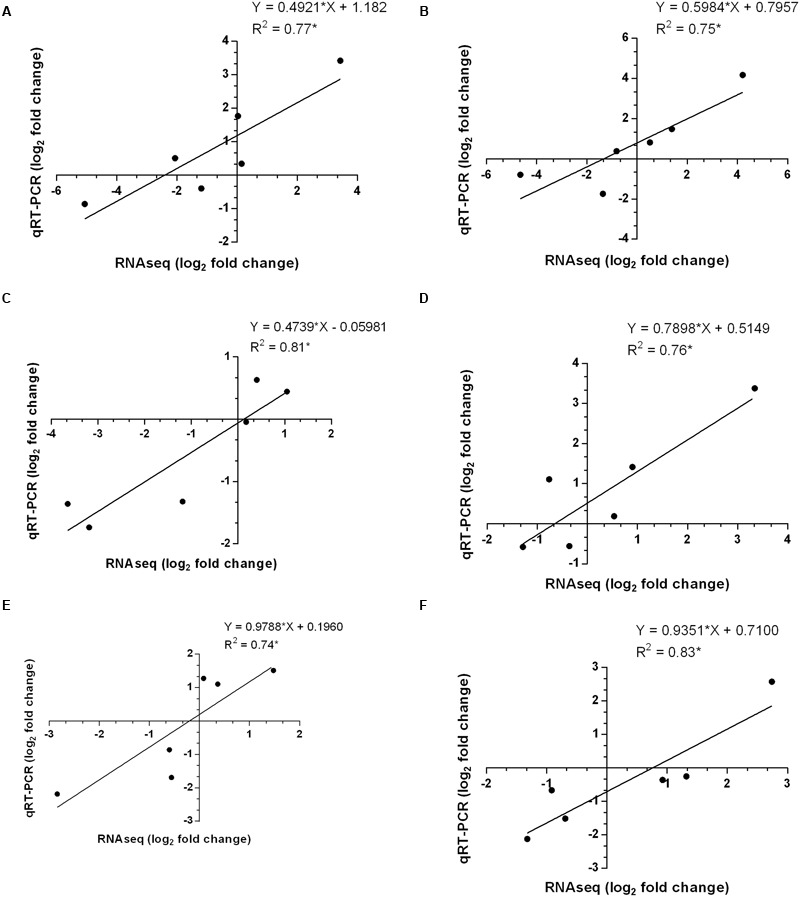
**Correlations determined between differential gene expression patterns of selected genes among transcriptomes data and qRT-PCR in different tissues of *P. kurroa* accessions; (A)** PKS-1 vs. PKS-4, **(B)** PKS-1 vs. PKS-5, **(C)** PKS-1 vs. PKS-21, **(D)** PKST-3 vs. PKST-5, **(E)** PKST-3 vs. PKST-16 and, **(F)** PKST-3 vs. PKST-18. *R*^2^ = Coefficient of determination.

Finally, the correlations between differential gene expression patterns of six selected genes obtained from transcriptomes data and qRT-PCR were determined and we observed coefficient of determination (*R*^2^) ≥ 0.75 which indicates a good positive correlation (**Figure 7**). It was thus evident from the results that gene expression patterns investigated by qRT-PCR were corroborated with those obtained by RNA-seq analysis, thereby supporting the reliability of our transcriptomes data.

## Discussion

The metabolic network of picrosides production is overwhelmingly complex as multiple pathways contribute to their biosynthesis in *P. kurroa*. In recent years, the major attention was paid to elucidate the metabolic flux dynamics in P-I production through the use of various inhibitors and precursors specific to the enzymes catalyzing critical steps in its biosynthesis (Shitiz et al., 2015; Kumar et al., 2016b). However, the biosynthesis of P-II remains untapped since it is not produced in *P. kurroa* plants grown *in vitro* (Sood and Chauhan, 2010). Moreover, the shoots of *P. kurroa* plants grown in natural habitats exhibited only P-I while stolons contained both P-I and P-II (Pandit et al., 2013a). Thus, to investigate the metabolic basis of P-II biosynthesis, we have employed a strategy utilizing natural genetic diversity existing for the production of P-II among *P. kurroa* accessions collected from different geographic locations of North-Western Himalayas, India. Moreover, the genes/enzymes information for most of the enzymatic steps has been obtained through generating and mining NGS transcriptomes from differential content phenotypes of *P. kurroa*. The differential transcriptomes provided greater insights not only into the genes involved in biosynthetic pathways but also molecular mechanisms that regulate the contents of picrosides in *P. kurroa*. In the present study, the analysis of stolon tissues from different *P. kurroa* accessions revealed prominent increase in P-II content upon reduction in P-I level indicating that both P-I and P-II biosynthesis skewed from a common metabolic node. It is likely since VA, the immediate precursor of P-II; is supposed to be formed from either PA or FA, both are derived from shikimate/phenylpropanoid pathway which also produces CA, the immediate biosynthetic precursor of P-I (Funk and Brodelius, 1992; Kumar et al., 2013, 2016b).

**FIGURE 8:**
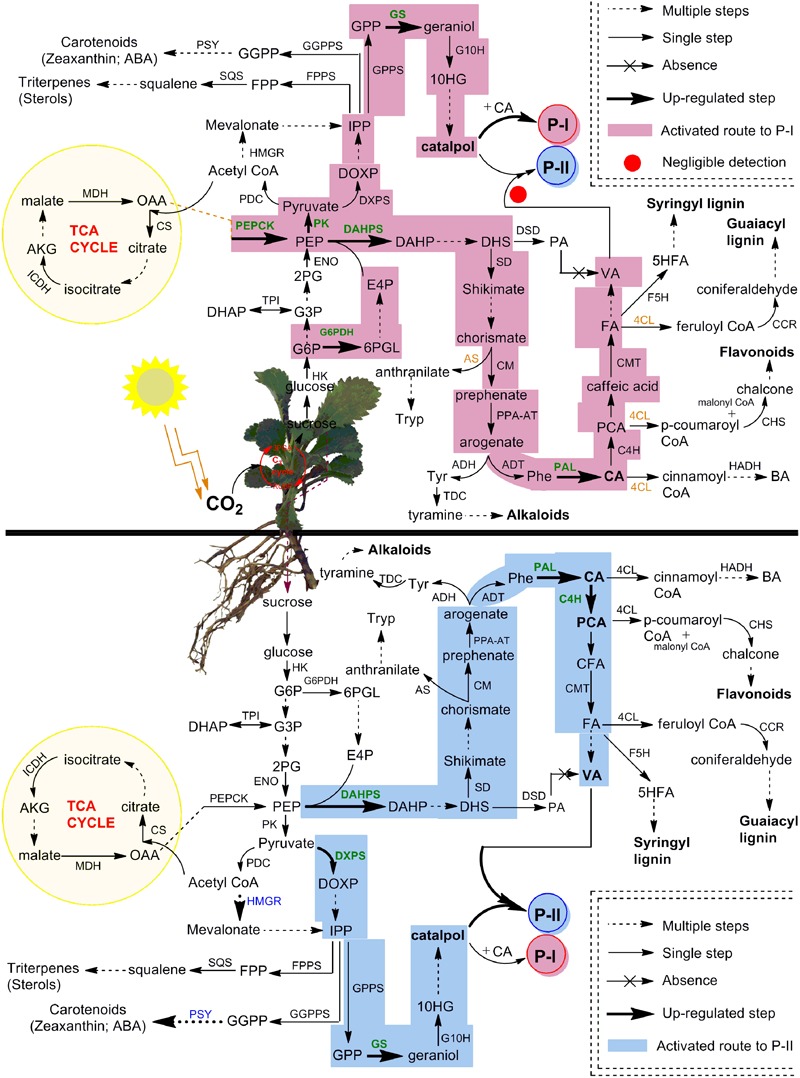
**Complete framework of metabolic network depicted P-I and P-II biosynthesis in different compartments of *P. kurroa*.** The established routes of P-I and P-II production were presented with different colors to highlight the modulations in shoots and stolons tissues of *P. kurroa*. Up-regulated gene expressions were highlighted with bold and green color. The abbreviations are elaborated in Supplementary Table 3.

The shikimate/phenylpropanoid pathway produces numerous branchpoints such as chorismate, arogenate, CA, *p*-CA, caffeic acid and FA, which act as precursors for the formation of phenylpropanoids, alkaloids, flavonoids, lignins, and picrosides, i.e., P-I and P-II (Tohge et al., 2013; Alvarez et al., 2016; Kumar et al., 2016b). The chorismate is involved in the formation of both tryptophan and arogenate metabolites (Kumar et al., 2016b), the later one on the other hand, can be converted into tyrosine and phenylalanine, the branchpoints for the formation of alkaloids and phenylpropanoids, respectively (Pascual et al., 2016; Schlager and Drager, 2016). The CA formed from phenylalanine acts as immediate precursor to P-I and also produces various phenylpropanoids *via* the formation of *p*-CA (Kumar et al., 2016b; Rigano et al., 2016). Moreover, the CoA pools generated from CA, *p*-CA, caffeic acid, and FA can deviate P-II biosynthesis by directing the flux of shikimate/phenylpropanoid pathway toward the formation of flavonoids and lignins (Alvarez et al., 2016; **Figure 8**).

Therefore, it is crucial to monitor dynamic profiles of intermediate metabolites in shikimate/phenylpropanoid pathway to get deep insight for metabolic switches of P-II biosynthesis. To address this, we have analyzed stolon tissues of four *P. kurroa* accessions based on their varying P-II content ranged from minimum to maximum (1.36–2.65%). The results observed in this study revealed statistically significant increase in VA content among high P-II content stolon tissues (PKST-3 and PKST-5) compared to stolons possessing low P-II content (PKST-16 and PKST-18) which established that VA is associated with P-II biosynthesis. This result is consistent with the proposed hypothesis that VA is the immediate precursor of P-II biosynthesis (Kumar et al., 2013). It is noteworthy that analysis of CA and *p*-CA metabolites content in selected stolon tissues revealed the flux direction of shikimate/phenylpropanoid pathway through to the CA step leading to the biosynthesis of *p*-CA and P-I as CA is located at the branching point between P-I and *p*-CA production (Kumar et al., 2016b). Interestingly, 87% CA was converted to *p*-CA in PKST-5 (P-1, 0.08%; P-II, 2.19%), 44.4% in PKST-3 (P-1, 0.17%; P-II, 2.65%), 21.1% in PKST-16 (P-1, 1.05%; P-II, 1.52%) and 8.2% in PKST-18 (P-1, 1.32%; P-II, 1.36%). This implies that with increase in the P-I content, the flux of shikimate/phenylpropanoid pathway limits through to the CA step to *p*-CA while increase in the P-II content directs the flux of shikimate/phenylpropanoid pathway through to the CA to *p*-CA leading to the enhanced P-II production in *P. kurroa* (**Figure 8**). This statement is also supported by comparative transcriptome analysis of selected stolon tissues which showed high positive correlation of gene encoding cinnamic acid-4-hydroxylase (0.60) with P-II content compared to gene encoding phenylalanine ammonia lyase enzyme which showed PCC of 0.28 with P-II content (**Figure 5C**). The cinnamic acid-4-hydroxylase is an enzyme that catalyzes the conversion of CA to *p*-CA while phenylalanine ammonia lyase catalyzes the conversion of phenylalanine to CA, the immediate precursor of the P-I biosynthesis (de Jong et al., 2015; Kumar et al., 2016b). Therefore, based on this analysis, we have hypothesized that CA is the common metabolic node between P-I and P-II biosynthesis in *P. kurroa* rather than 3-dehydroshikimate which serves as the branch point between PA and shikimate production; the metabolites also supposed to be the common nodes for P-I and P-II biosynthesis (Shitiz et al., 2015).

The analysis of FA, however, did not show statistically significant variations among selected stolon tissues. Albeit, increased VA content was observed in PKST-3 and PKST-5; stolons having high P-II content, compared to PKST-16 and PKST-18; low P-II content stolons. Keeping in view that FA is a canonical intermediate metabolite in the biosynthesis of guaiacyl- and syringyl lignin’s in addition to its proposed role in VA production, the results observed in this study might indicate that flux of FA is directed toward both VA and P-II in PKST-3 and PKST-5, whereas maximum flux of FA might deviate to lignin biosynthesis in PKST-16 and PKST-18. This statement is in agreement with correlation map constructed between P-II content and transcripts abundance values (FPKM) which revealed negative correlation of gene encoding caffeic acid-3-*O*-methyltransferase (CMT) with P-II content (-0.63) followed by a strong positive correlation of *CMT* through a PCC value of 0.82 with ferulic acid-5-hydroxylase (F5H), an enzyme that catalyzes the conversion of ferulic acid to 5-hydroxy ferulic acid, which represents as a precursor for syringyl lignin biosynthesis (Wagner et al., 2015). Consequently, it is apparent that up-regulated transcript level of CMT; an enzyme catalyzing the conversion of caffeic acid to FA accompanied by F5H, might be linked to the activation of lignin biosynthesis in PKST-16 and PKST-18. Trabucco et al. (2013) also reported the reduced total lignin content upon down-regulation of gene encoding CMT enzyme in *Brachypodium distachyon*. As a whole, the analysis of stolon tissues with natural variations for P-II content hypothesized that P-II is produced via shikimate/phenylpropanoid pathway through degradation of FA to VA, which finally integrates with catalpol (CAT) to produce P-II in *P. kurroa* (**Figure 8**).

Previous studies have reported that shoots of *P. kurroa* are independent biosynthetic tissues for P-I, a secondary metabolite produced by the combination of CA and catalpol (Kumar et al., 2016b). Nevertheless, current study established stolons of *P. kurroa* as autonomous biosynthetic machinery for P-I and P-II, both share a common biosynthetic pathway, it is inspiring to comprehend the negligence of P-II biosynthesis in *P. kurroa* shoots. To address this, the shoot tissues of four *P. kurroa* accessions based on their varying P-I content ranged from minimum to maximum (0.13–2.31%) were selected for analysis of *p*-CA, CA, FA, and VA with the aim to investigate the fate of shikimate/phenylpropanoid pathway in *P. kurroa* shoots. The analysis of shoot tissues of four *P. kurroa* accessions for CA and *p*-CA revealed that increased P-I production limits flux of CA toward *p*-CA biosynthesis. It is likely since CA shares a common biosynthetic node for P-I and *p*-CA (Kumar et al., 2016b). In contrast, the analysis of FA revealed slight significant variation between the selected shoots and also showed a high positive correlation by PCC of 0.85 with *p*-CA. However, *C4H* and chalcone synthase (*CHS*) showed a strong positive correlation with PCC of 0.90 compared to PCC of 0.23 between *C4H* and *CMT* (**Figure 5D**). CHS is an enzyme that catalyzes the formation of naringenin chalcone; the later one serves as a starting metabolite for flavonoids biosynthesis (Zuk et al., 2016). Therefore, this finding underscores that phenylpropanoid pathway might allocate the flux of *p*-CA to produce both FA and flavonoids in *P. kurroa* shoots. This statement is in agreement with previous study which revealed that silencing of gene encoding hydroxycinnamoyl-CoA shikimate/quinate hydroxycinnamoyltransferase enzyme (HCT) involved in lignin biosynthesis, re-routed the flux of phenylpropanoid pathway into flavonoids through CHS activity (Besseau et al., 2007). Interestingly, negligible levels of VA were observed in all the selected shoot tissues which implies that P-II biosynthesis arrests in the downstream steps of FA, which possibly limits the VA supply.

Therefore, *p*-CA, FA, and PA were exogenously applied along with mixture of VA and catalpol in tissue culture conditions not only to establish the course of P-II biosynthesis but also to produce P-II in *P. kurroa* shoots. The results revealed significant enhancement of VA content in FA fed shoots compared to shoots without treatment. This observation further ascertained that FA is associated with VA production rather than PA which is previously proposed to be the precursor for VA biosynthesis (Kumar et al., 2013; Shitiz et al., 2015). Moreover, significant enhancement of VA content in VA+CAT fed shoots over untreated control might indicate it’s accumulation due to exogenous application. This was also supported by the absence of VA content observed in the MS media left after the collection of VA+CAT fed shoots. Upon analysis of P-II content, minor detection was only observed in VA+CAT fed shoots which indicate that exogenous application of VA+CAT, both immediate precursors of P-II biosynthesis, might stimulate its production in *P. kurroa* shoots. Therefore, different concentrations of VA+CAT were further tested with the aim to observe the progressive increase of P-II content in *P. kurroa* shoots. Unfortunately, we did not observe significant increment in P-II content among different VA+CAT fed shoots which is possibly due to low activity of a probable acyltransferase that catalyzes the esterification of VA and CAT to produce P-II. However, we have not observed the activity of probable acyltransferase as it is not discovered yet. It is striking that P-II was not taken up by the *P. kurroa* plants upon its exogenous introduction in purified form. This was clearly demonstrated from the negligible level of P-II observed in shoots fed with P-II whereas its substantial content was detected in the MS media left after the collection of P-II fed shoots. This finding suggests that *P. kurroa* plants are unable to take P-II in its ready form and subsequently they are dependent on its endogenous production.

Taken together, the findings of this study shed light on metabolic modulations underlying the biosynthesis of P-I and P-II in *P. kurroa*. It offers convincing evidences that independent mechanisms control the biosynthesis of P-I and P-II in both compartments of *P. kurroa*, i.e., shoots and stolons. The intermediates of shikimate/phenylpropanoid pathway produced in stolons guided the flux toward P-II biosynthesis via degradation of FA to produce VA, the immediate precursor of P-II. In contrast, flux of precursors deviate from the non-canonical view of P-II biosynthesis in shoot tissues of *P. kurroa*. Theoretically, we have finally illustrated the basis of P-II negligence in shoot compartment that provides impetus for the future investigation of an enzyme catalyzing the conversion of VA and CAT to produce P-II, a task that can rewrite the P-II production in shoots of *P. kurroa*.

## Author Contributions

VK conducted tissue culture experiments. VK conducted molecular experiments and HPLC analysis. AB performed transcriptomes data analysis and VK conducted correlation analysis. VK and RC conceived and designed research. VK, AB, and RC analyzed data. VK and RC wrote the manuscript. All authors read and approved the manuscript.

## Conflict of Interest Statement

The authors declare that the research was conducted in the absence of any commercial or financial relationships that could be construed as a potential conflict of interest.

## Footnotes

1 http://michaelrthon.com/runiprscan/

2 http://14.139.240.55/NGS/download.php
