# Supplementary material for: Modular Design of Picroside-II Biosynthesis Deciphered through NGS Transcriptomes and Metabolic Intermediates Analysis in Naturally Variant Chemotypes of a Medicinal Herb, Picrorhiza kurroa
Source: Front Plant Sci. 2017 Apr 11;8:564. doi: 10.3389/fpls.2017.00564 (PMC5387076; doi:10.3389/fpls.2017.00564)
Supplement: Supplementary file 1 [file Table_1.DOC]

**Supplementary Table 1.** Details of *P. kurroa* accessions collected from different geographical locations of North-Western Himalaya, India (Shitiz et al. 2016)

| S. No. | Accession | Location/District | Altitude | Latitude | Longitude |
| --- | --- | --- | --- | --- | --- |
| 1 | PK-1 | Hudan Bhatori/Chamba | 3620 | 33006’27.1” N | 76029’171.1” E |
| 2 | PK-2 | Bhuri/Kinnaur | 3330 | 31030’01.3” N | 78056’34.5” E |
| 3 | PK-3 | Dhel/Kullu | 3597 | 31045.412” N | 77027.680” E |
| 4 | PK-4 | Teita/Chamba | 3590 | 32°31’28” N | 76°31’01” E |
| 5 | PK-5 | Moral Danda/Shimla | 3354 | 31018’23.6” N | 77045’02.1” E |
| 6 | PK-14 | Yungpa/Kinnaur | 3440 | 34040’5.1” N | 78001’12.0” E |
| 7 | PK-16 | Salam Tith/Chamba | 3440 | 33059.142” N | 77011.173” E |
| 8 | PK-18 | Sural Bhatori/Chamba | 3323 | 33008’41.6” N | 76027’49.4” E |
| 9 | PK-21 | Pattal/Chamba | 3245 | 32057.321” N | 76018.417” E |
| 10 | PK-26 | Shringul Tung/Shimla | 3307 | 32°14’59” N | 78°06’48” E |
