# Supplementary material for: Modular Design of Picroside-II Biosynthesis Deciphered through NGS Transcriptomes and Metabolic Intermediates Analysis in Naturally Variant Chemotypes of a Medicinal Herb, Picrorhiza kurroa
Source: Front Plant Sci. 2017 Apr 11;8:564. doi: 10.3389/fpls.2017.00564 (PMC5387076; doi:10.3389/fpls.2017.00564)
Supplement: Supplementary file 2 [file Table_2.DOC]

**Supplementary Table 2. Details of primers used for qRT-PCR analysis of selected genes**

| Genes | Primer sequence | Fragment size (bp) | Annealing temperatures (^0^C) |
| --- | --- | --- | --- |
| 26S | FP 5’-CACAATGATAGGAAGAGCCGAC-3’  RP 5’-CAAGGGAACGGGCTTGGCAGAATC-3’ | 500 | 58 |
| DAHPS | FP 5’-ACACCATTAAAGCTCCTTGT-3’  RP 5’-TAACAGTCTGAGATCCACCA-3’ | 171 | 59 |
| CMT | FP 5’-GAAGATGCTCCTTCTTATCC-3’  RP 5’-AACACTCGACCAGAATCAC-3’ | 187 | 53 |
| PAL | FP 5’-GCAAGATAGATACGCTCTAA-3’  RP 5’-GTTCCTTGAGACGTCAAT-3’ | 136 | 49 |
| C4H | FP 5’-GCAACATTGATGTTCTCAAC-3’  RP 5’-TCCAGCTCTTCAAGGACTAT-3’ | 169 | 53 |
| HK | FP 5’-ATGCTCCTTACCTACGTTCA-3’  RP 5’-TCCTAACTGAACCCTCAAGA-3’ | 108 | 52 |
| G10H | FP 5’-TATCGAGCTTTTCAGTGGAT-3’  RP 5’-GATGTGAGTCCTGTCGATTT-3’ | 136 | 52 |
