# Supplementary material for: Modular Design of Picroside-II Biosynthesis Deciphered through NGS Transcriptomes and Metabolic Intermediates Analysis in Naturally Variant Chemotypes of a Medicinal Herb, Picrorhiza kurroa
Source: Front Plant Sci. 2017 Apr 11;8:564. doi: 10.3389/fpls.2017.00564 (PMC5387076; doi:10.3389/fpls.2017.00564)
Supplement: Supplementary file 3 [file Table_3.DOC]

**Supplementary Table 3.** Abbreviations in Figure 9

| **Abbreviations** | **Name** | **Abbreviations** | **Name** |
| --- | --- | --- | --- |
| G-6-P | Glucose-6-phosphate | 10HG | 10-hydroxygeraniol |
| ABA | Abscisic acid | PK | Pyruvate kinase |
| PSY | Pytoene synthase | ICDH | Isocitrate dehydrogenase |
| DHAP | Dihydroxyacetonephosphate | MDH | Malate dehydrogenase |
| G-3-P | Glyceraldehyde-3-phosphate | AKG | α-keto glutarate |
| SQS | Squalene synthase | G6PDH | Glucose-6-phosphate dehydrogenase |
| OAA | Oxaloacetate | P-I | Picroside-I |
| 2-PG | 2-phosphoglycerate | P-II | Picroside-II |
| PEP | Phosphoenolpyruvate | CA | Cinnamic acid |
| 6-PGL | 6-phosphogluconolactone | AS | Anthranilate synthase |
| FPP | Farnesyl pyrophosphate | DAHPS | 3-Deoxy-D-arabinoheptulosonate 7-phosphate synthase |
| FPPS | Farnesyl pyrophosphate synthase | Tryp | Tryptophan |
| GGPP | Geranylgeranyl pyrophosphate | ADH | Arogenate dehydrogenase |
| GGPPS | Geranylgeranyl pyrophosphate synthase | SD | Shikimate dehydrogenase |
| E-4-P | Erythrose-4-phosphate | Tyr | Tyrosine |
| DOXP | 1-deoxy-D-xylulose-5-phosphate | TDC | Tyrosine decarboxylase |
| 3-PGA | 3-phosphoglycerate | C4H | Cinnamic acid-4-hydroxylase |
| RUBP | Ribulose 1,5-bisphosphate | CM | Chorismate mutase |
| CO_2_ | Carbon dioxide | PPA-AT | Prephenate aminotransferase |
| ENO | Enolase | ADT | Arogenate dehydratase |
| PDC | Pyruvate dehydrogenase complex | PAL | Phenylalanine ammonia lyase |
| IPP | Isopentenyl pyrophosphate | DXPS | 1-deoxy-D-xylulose-5-phosphate synthase |
| DSD | Dehydroshikimate dehydratase | PCA | p-coumaric acid |
| DAHP | 3-Deoxy-D-arabinoheptulosonate 7-phosphate | 4CL | 4-coumarate CoA ligase |
| PRCA | Protocatechuate | BA | Benzoic acid |
| DHS | 3-dehydroshikimate | HADH | 3-hydroxyacyl CoA dehydrogenase |
| CHS | Chalcone synthase | CMT | Caffeic acid-3-O methyltransferase |
| Phe | Phenylalanine | FA | Ferulic acid |
| CCR | Cinnamoyl CoA reductase | F5H | Ferulic acid-5-hydroxylase |
| GPP | Geranyl pyrophosphate | 5HFA | 5-hydroxyferulic acid |
| VA | Vanillic acid | HMGR | Hydroxymethylglutaryl-CoA reductase |
| HK | Hexokinase | GPPS | Geranyl pyrophosphate synthase |
| TPI | Triosephosphate isomerase | GS | Geraniol synthase |
| G10H | Geraniol-10-hydroxylase | TCA | Tricarboxylic acid |
